# Balancing stability and function: impact of the surface charge of SARS-CoV-2 Omicron spike protein

**DOI:** 10.1038/s44298-025-00104-1

**Published:** 2025-04-01

**Authors:** Daniel Lauster, Rainer Haag, Matthias Ballauff, Andreas Herrmann

**Affiliations:** 1 Institute of Pharmacy, Biopharmaceuticals, Freie Universität Berlin, Berlin, Germany; 2 Institute of Chemistry and Biochemistry, Freie Universität Berlin, Berlin, Germany

**Keywords:** SARS-CoV-2, Viral evolution

## Abstract

The ectodomain of the Omicron SARS-CoV-2 spike has an increased positive surface charge, favoring binding to the host cell surface, but may affect the stability of the ectodomain. Thermal stability studies identified two transitions associated with the flexibility of the receptor binding domain and the unfolding of the whole ectodomain, respectively. Despite destabilizing effects of some mutations, compensatory mutations maintain ECD stability and functional advantages thus supporting viral fitness.

During and after the Covid19 pandemic subsided, SARS-CoV-2 variants of concern (VoCs) evolved in a parallel and in successive manner. These VoCs were characterized by mutations in their various proteins, including the homotrimeric envelope spike glycoprotein (S). The ectodomain (ECD) of the S protein is central to the attachment of the virus to the host cell and to the initiation of fusion of the envelope membrane either with the plasma membrane or the endosomal membrane following endocytic uptake of the virus^1^.

A wild type (wt; Wuhan) S monomer consists of a total of 1273 amino acid residues, of which 1208 form the ectodomain. Each monomer consists of two subunits, S1 and S2. The distal S1 subunit contains the N-terminal domain (NTD) and the host cell receptor binding domain (RBD) (Fig. 1). The S2 subunit anchors the entire protein to the viral envelope via a hydrophobic transmembrane domain. The ectodomain comprises the S1 and part of the S2 subunits, including the heptad repeats HR1 and HR2. Infection requires both proteolytic cleavage of the two subunits by a furin-like protease and cleavage of a conserved site on the S2 subunit immediately adjacent to the fusion peptide by the type II transmembrane serine protease (TMPRSS2)^1–3^. In the prefusion state, the RBD switches between the up and down conformations. In the up (open) conformation, it binds the host cell receptor.

**Fig. 1:**
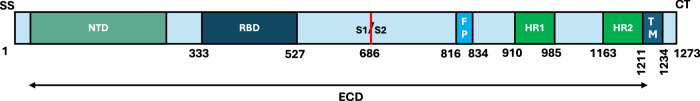
Organization of the S spike protein of SARS-CoV-2 S. SS—signal sequence, NTD—N terminal domain, RBD—receptor binding domain, S1/S2—furin cleavage site, FP—fusion peptide, HR1 and HR2—heptad repeat sequences 1 and 2, TM—transmembrane domain, CT—cytosolic (intraviral) domain. The signal sequence is removed by cellular signal peptidases in the lumen of the endoplasmic reticulum of the host cell.

ACE2 was identified as the host cell-specific receptor for SARS-CoV-2^1^, as already known from SARS-CoV-1. S1 binds to ACE2 with a specific receptor binding motif (RBM) of the RBD covering residues 333 to 527 (residue numbers given correspond to the original Wuhan S sequence). However, without questioning the prominent role of ACE2, other specific binding receptors have been identified^4–7^. While the mutations in the ECD of the S proteins of the various VoCs play an important role in escape from neutralizing antibodies^8,9^, they are also often favorable for binding to ACE2^10–13^.

However, evidence has been accumulated that highly charged heparan sulfate proteoglycans (HSPG) in the extracellular matrix serve as key initial binding sites for SARS-CoV-2^14–19^. The negatively charged heparan sulfate (HS) chains electrostatically interact with positively charged amino acids on the S protein’s ECD surface^20^. Mutations in the ECD have increased these positively charged amino acids in Delta and even more so in Omicron BA.1 (B.1.1529.1) compared to the wild type (see Table 1), significantly enhancing the S domain’s positive surface potential and its affinity for HSPG^13,20–25^.

**Table 1:** Quantitative differences in positively and negatively charged amino acids of the whole ectodomain (ECD) and only of the RBD between wt and subsequent VoCs (for details see also Supplementary Table 1)

Strain	ECD			RBD		
	# Δ (R + K)	# Δ (D + E)	Change of Net charge	# Δ (R + K)	# Δ (D + E)	Change of Net charge
Delta	4	−3	( + )7	2	0	( + )2
BA.1	8	−1	( + )9	3	0	( + )3
BA.2	5	−2	( + )7	2	−1	( + )3
BA.2.75	5	−2	( + )7	1	−2	( + )3
XBB.1.5	4	−1	( + )5	1	−2	( + )3
XBB.1.16	4	−2	( + )6	1	−2	( + )3
EG.5	4	−1	( + )5	1	−2	( + )3
HK.3	4	−1	( + )5	1	−2	( + )3
JN.1	7	−2	( + )9	3	−1	( + )4
KP.2	7	−2	( + )9	2	−1	( + )3
KP.3	8	−1	( + )9	3	0	( + )3
BA.3	6	−2	( + )8	3	−1	( + )4
BA.4	5	−3	( + )8	2	−1	( + )3

# Δ (R + K) and # Δ (D + E) refers to the difference in the number of charged amino acids between wt and VoCs. The compensation of positively charged residues on the spike protein is > 0, and a loss of D or E results in an uncharged amino acid. As a consequence, the compensation of positive countercharge on the S protein is lost when D or E is mutated, resulting in an increase ( + 1) in the net charge. The change of in net charge is given by:

$(+1)\#\Delta (R+K)-(-1)\#(D+E)$ where ( + 1) and (−1) are net charges of amino acids

(Sequences are taken from: wt: www.ncbi.nlm.nih.gov/protein/YP_009724390.1; BA.1*: Slovenia/178530/2022; BA.2*: Norway/1831/2022; BA.2.75*: Croatia/HZJZ_1619/2022; XBB.1.5*: Denmark/DCGC-662916/2023; XBB.1.16*: Denmark/DCGC-662929/2023; EG.5*: Austria/AGES-AZ-1662675/2023; HK.3*: France/OCC-CHU-TLS-6004314441/2023; JN.1: https://outbreak.info/situation-reports?xmin=2023-12-22&xmax=2024-06-22&pango=JN.1; KP.2: https://outbreak.info/situation-reports?xmin=2023-12-22&xmax=2024-06-22&pango=KP.2; KP.3: https://outbreak.info/situation-reports?xmin=2023-12-22&xmax=2024-06-22&pango=KP.3; BA. 3: https://outbreak.info/situation-reports?xmin=2023-12-11&xmax=2024-06-11&pango=BA.3; BA.4*: Pakistan/NIH-B69-S5/2022;

*BA.1, BA.2, BA.2.75, XBB.1.5., XBB.1.16, EG.5, HK.3 BA.4 are taken from https://nextstrain.org/ncov/gisaid/global/6m?s=hCoV-19/.

However, an increased surface potential can be unfavorable for protein stability. It has long been established that higher surface charge correlates with a decrease in the denaturation temperature of most proteins^26,27^, with many exhibiting maximum thermal stability near their isoelectric point^28,29^. The decreased stability due to an increased number of charges can have significant biological consequences^30^. Typical of class I fusion proteins, the prefusion state of S proteins is metastable and undergoes an irreversible conformational change to mediate fusion between the viral envelope and the target membrane. Increased surface charge can destabilize this prefusion state, potentially triggering an uncontrolled transition to the post-fusion state. In this study, we screened post-BA.1 Omicron variants for charged amino acid mutations in the ECD and assessed their potential impact on ECD stability. To this end, we reviewed here reports on the thermal behavior and transitions of the ECD and the RBD to assess whether their results suggest a reduced stability of the ECD.

## Charged amino acids in the ectodomain of SARS-CoV-2 Omicron variants

**Fig. 2:**
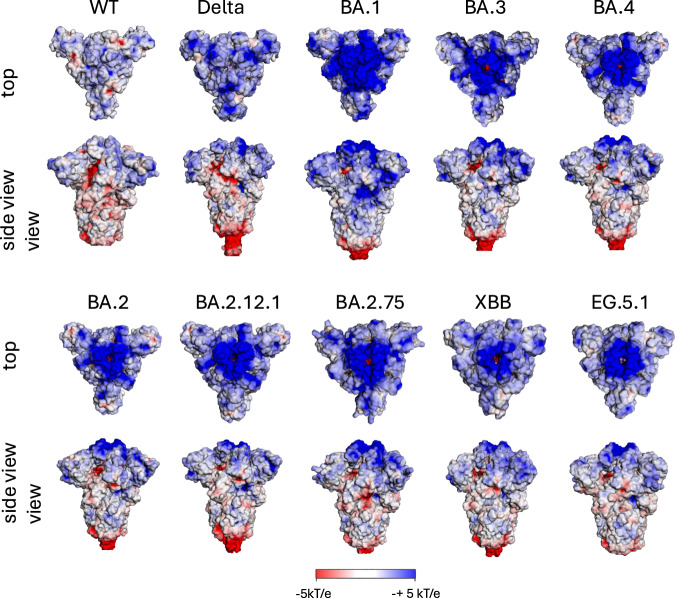
Surface potential of the S ectodomain. Surface potential of the S ectodomain of the wt and of several variants of concern (VoCs) in the closed state of RBDs at pH 7.0 (pdb: 7QUS (wt); 7SBK (Delta); 7WK2 (BA.1); 7XIX (BA.2); 7XNS (BA.2.12.1); 7YQU (BA.2.75); 8PSD (XBB.1); 8IOS (EG.5.1); 7XIY (BA.3); 7XNS (BA.4)). Note that for the purpose of stability the furin cleavage site in the stem region has been removed for the various spike proteins as usually done for 3D structure determination and other experimental setups. The surface potential of these proteins was calculated and visualized by using the software ‘Adaptive Poison-Boltzmann-Solver’ (APBS)^69^ via the ABPS server (https://server.poissonboltzmann.org) or alternatively via the plugin of PyMOL program (version3.0.2.; license #51548).

The distal region of the ECD of the S spike protein in BA.1 showed a significant accumulation of exposed positively charged amino acids. As we have shown previously, this resulted in a significant increase in the surface potential of the distal head domain of the S trimer when compared to the wt SARS-CoV-2^20^. In contrast, there were only minor alterations in the rather neutral surface potential of the ECD’s stem region. Similar changes in charged amino acids and in the surface potential pattern we observed in the ECD of BA.2 and Omicron variants BA.3 and BA.4^13^ shown in Fig. 2.

Analysis of more recent Omicron variants now shows that this pattern has been maintained (Fig. 2). The XBB variants are recombinant strains of the two BA.2 sublines BJ.1 and BM.1.1.1, EG.5/EG.5.1 is a descendant lineage of XBB.1.9.2, which has the same spike amino acid profile as XBB.1.5, and HK.3 (also XBB.1.9.2.5.1.1.3) has evolved from EG.5.1^31^. The phylogeny of the variants used here is shown in Supplementary Fig. 1 (generated via nextstrain.org/sars-cov-2^32^).

The increased positive surface potential of the S protein of the recent variants is also restricted to the distal head domain. A further increase in the positive surface potential compared to BA.1 and BA.2 is not detectable. The latter is also reflected in the composition of ECD regarding charged amino acids. The quantitative differences between the wt and the following VoCs in positively and negatively charged amino acids are shown in Table 1 (sequence changes of charged amino acids between wt and Omicron VoCs see Supplementary Table 1). It is obvious that there was no further increase in the net charge of the recent Omicron compared to BA.1 and BA.2. The net charge of the currently dominant variants JN.1, KP.2, and KP.3 is comparable to that of BA.1. JN.1 is a parent lineage of BA.2.86, KP.2 and KP.3 are descendants of JN.1

The slight changes in the number of charged amino acids in the subsequent Omicron VoCs do not significantly influence the pattern of the surface potential of the S protein being still comparable to that of the BA.1 and BA.2 strains (Fig. 2). This is particularly evident in the distal part of the domain.

## Thermal transitions of the ectodomain of S spike protein

Several studies have measured the thermal stability of the S trimer ectodomain and/or in a few cases of the RBD (for an overview see Supplementary Table 2). Only one study used Differential Scanning Calorimetry (DSC). In most cases, thermal stability was measured by Differential Scanning Fluorometry (DSF)^33,34^. Either the intrinsic fluorescence or that of the dye SYPRO Orange interacting with the protein was used for detection by DSF (see Supplementary Table 2). In DSF, the temperature dependence of the ratio of fluorescence at two different wavelengths (330 nm vs. 350 nm) is measured. The local maxima derived from the first derivative of the corresponding curve, termed as melting temperatures (Tm), are used to characterize the thermal behavior of trimers or the RBD. Typically, the temperatures of two consecutive inflection points—Tm_1_ < Tm_2_—have been reported for the ECD. A few DSF studies also reported a third turning point at Tm_3_ (Tm_2_ < Tm_3_). The data are summarized in Supplementary Table 2.

**Fig. 3:**
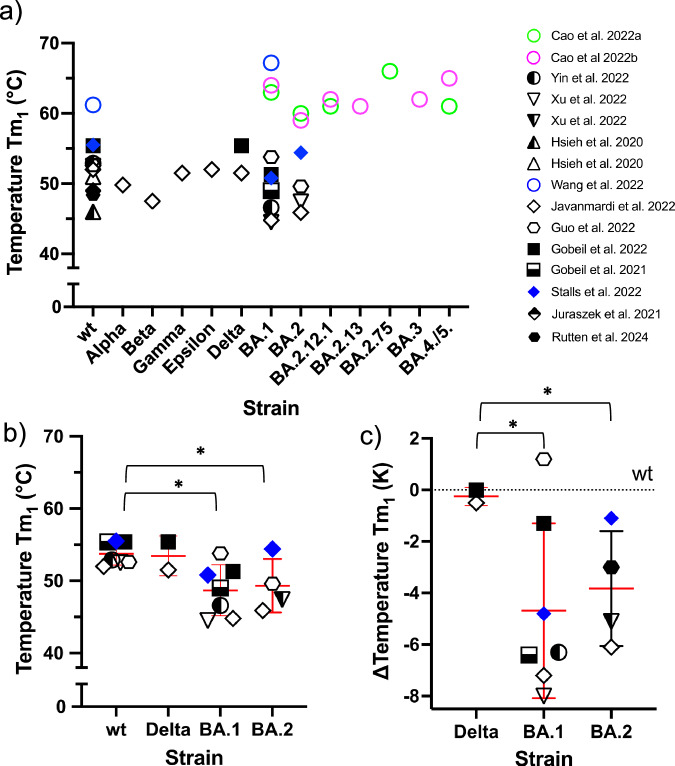
The first transition temperature Tm_1_ of the S ectodomain. Measurements refer to DSF studies, with the exception of those by Juraszek et al. ^35^ and Rutten et al. ^36^ (DSC). **a** The first melting temperature Tm_1_ is shown. Proline mutations for stabilization: Filled symbols—no proline mutations; half-filled symbols—two prolines; empty symbols—six prolines (see text for explanation). Tm_1_ (**b**), derived from (**a**) and (**c**) the difference ΔTm_1_ between the Tm_1_ of the wt and the Tm_1_ values of the variants of concern, as measured in the same study. Consequently, only studies in which the wt values were measured in addition to the VoCs were included in the analysis. Furthermore, only VoCs for which at least two independent studies were available were included. Mean and standard deviation are presented. Statistical analysis was performed on the data presented in (**b**). Although the Kolmogorov-Smirnov test indicated that the distribution of the data for strains wt, BA.1, and BA.2 followed a normal distribution (*P* = 0.05), an additional analysis was performed using the non-parametric Kruskal-Wallis test in conjunction with one-way ANOVA. At a significance level of 0.05 (*), both tests showed a statistically significant difference in the Tm_1_ values between BA.1 and BA.2 and the wt value. It should be noted that the statistical analysis of the published data on the Delta variant is based on only two values and is therefore limited in its validity.

**Fig. 4:**
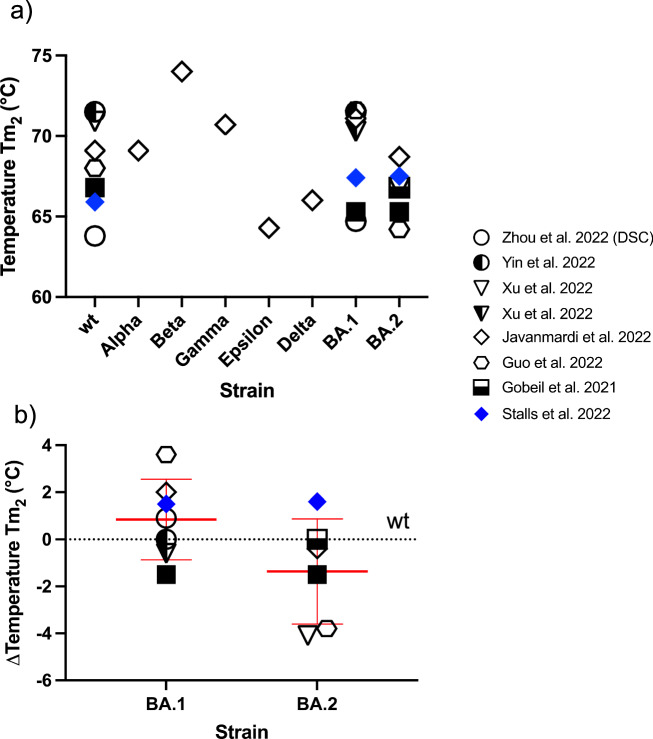
The second transition temperature Tm_2_ of the S ectodomain. **a** Summary of studies providing data for the second transition temperature Tm_2_ of the S ectodomain measured by DSF, with the exception of the DSC study by Zhou et al. ^37^ The latter provided data for wt and BA.1 (open circles). **b** DSF difference ΔTm_2_ between wt Tm_2_ and the Tm_2_ values of the variants of concern, as measured in the same study. Therefore, only studies in which the wt values were measured in addition to the VoCs were included in the analysis. Furthermore, only VoCs for which at least two independent studies were available were included. Proline mutations for stabilization: Filled symbols—no proline mutations; half-filled symbols—two prolines; empty symbols - six prolines (see text for explanation). Mean and standard deviation are shown.

As deduced from DSF measurements, Tm_1_ and Tm_2_ were typically between 45 and 55 °C and 64 °C and 70 °C respectively. In agreement with this, Juraszek et al. ^35^ and Rutten et al. ^36^ identified two discrete transitions for the wt using DSC at temperatures corresponding to Tm_1_ (Fig. 3a) and Tm_2_ (Fig. 4a) of the DSF measurement. The transition at Tm_2_ was much more intense than that of Tm_1_. Zhou et al. ^37^ reported only to a prominent transition at Tm_2_ for the wt spike protein. However, the DSC spectrum also shown indicates a rather small peak in the Tm_1_ region. Taken together, all the evidence points to two transitions, Tm_1_, and a second transition, Tm_2_ at higher temperatures. It is evident from the DSC data^35–37^ that the second transition at about 65 °C relates to the unfolding of the entire ECD and is characterized by a concomitantly high transition enthalpy of the order of 200 kcal/mol^37^. This transition therefore marks the upper limit of thermal stability. The first transition, however, must therefore be related to a transition within the ectodomain (see section on the thermal transition of RBD and Discussion).

The Tm_1_ data have been summarized in Fig. 3a. With the exception of the Alpha (B.1.1.7), Beta (B.1.351), Gamma (P1, B.1.1.28.1), Epsilon (B.1.427/29) and BA.2.13 variants^38^, for all other SARS-CoV-2 variants data of at least two independent reports are shown in Fig. 3a, with measurements for the wt being the most frequent.

As can be seen from Fig. 3a, the values for a given SARS-CoV-2 strain vary considerably in some cases, i.e., there is a large variation in data between studies. Nevertheless, the results indicate that the Tm_1_ of the ECD for the Omicron strains BA.1 and BA.2 is decreased compared to the wt. In Fig. 3b Tm_1_ values (taken from Fig. 3a) were selected that met two criteria: (i) studies in which wt Tm_1_ and Tm_1_ of VoCs were measured, and (ii) only those VoCs were included for which at least two independent studies were available. Based on this selection, only the thermal stability of the S protein between the wt, Delta, BA.1, and BA.2 variants can be compared. Statistical analysis showed that the Tm_1_ values of BA.1 and BA.2 were significantly different from that of the wt (see legend to Fig. 3). To further illustrate the differences between wt and the two Omicron variants, the differences between the Tm_1_ values of the wt and the VoCs (ΔTm_1_) for each study are shown in Fig. 3c.

Compared to wt, no change in ΔTm_1_ was observed for the S protein of the Delta variant. However, the significant decrease in ΔTm_1_ for the BA.1 and BA.2 variants, which averaging about 4 K, is noteworthy. Some studies have observed this decrease in ΔTm_1_ for both variants, while others have observed it only for BA.2 (Guo et al.)^39^. Still, others have reported this decrease only for BA.1 (Stalls et al.)^40^.

The only available data on S protein for VoCs subsequent to BA.2 are those in the two studies by Cao et al. ^41,42^ The results are indicative of a reduction in Tm_1_ for BA.2. Importantly, the data show that this reduction does not persist with subsequent strains, such as BA.2.13 and BA.2.75, but rather reverses. As no Tm_1_ values were given for the wt in these two studies, these results could not be taken into account in the representation of ΔTm_1_ in Fig. 3c, which is based on the values shown in Fig. 3b.

The published Tm_2_ values are shown in Fig. 4a. Once more, the Tm_2_ of the wt was the most frequently reported. Unfortunately, only measurements for BA.1 and BA.2 are available for VoCs. Only one study used DSC^37^, all others used DSF for the determination of Tm_2_. The DSC study was limited to the wt and BA.1. Comparison of the absolute values of Tm_2_ does not allow any conclusions to be drawn about possible differences between the wt and BA.1 and BA.2. Therefore, as with Tm_1_, a normalization to the Tm_2_ value of the wt S spike protein was made. It is evident from Fig. 4b that there is no change in the thermal stability of the two variants, BA.1 and BA.2. The aforementioned DSC study^43^ provided enthalpy values in addition to Tm_2_: a melting temperature of 63.8 °C and an enthalpy change (∆H) of 179 kcal/mol for wt, and 64.7 °C and 222 kcal/mol for BA.1, indicating an increased stability of the BA.1 ECD. Unfortunately, this is the only study that has provided enthalpy values.

The reliability of the results for the temperature values of the inflection points is largely determined by the scan rate of the measurements. Reliable statements require a low scan rate. As can be seen in Supplementary Table 2, the majority of measurements were carried out with a low scan rate in the range between 0.9 and 4.5 K/min. However, some studies were also carried out with a very high scan rate (30 K/min). Results obtained at such high scan rates must be treated with caution as to their accuracy, even though they are comparable to those obtained at lower rates, and also reflect differences between wt and BA.1/BA.2.

When comparing the thermal stability of the S protein in different VoCs, the effects of site directed mutations, which are often used to improve the stability of the prefusion conformation of the ECD, must be taken into account. Stabilization of the homotrimer ensemble of the ECD is typically achieved by the introduction of a C-terminal foldon trimerization motif. In addition, the furin cleavage site residues (aa 682-685) are mutated in order to stabilize the prefusion state of the spike protein (Hsieh et al.)^44^. However, few studies have focused solely on these stabilizing modifications^35,36,45,46^. Typically, the prefusion conformation is additionally stabilized by either with two proline or even with six proline insertions in the stem region, i.e., in the S2 subunit, of each monomer. In the first case, residues 986 and 987 are located between the central helix and the heptad repeat 1 (HR1)^44^, in the second case, four additional residues (aa 817, 892, 899, 942) are substituted by prolines. All cited references that investigated the ECD used the complete ECD, along with the stabilization modifications mentioned above. Typically, the TMD and thus the CT were cut off, as the TMD leads to aggregation of the S trimers due to its highly hydrophobic properties, e.g., in the form of rosettes. However, it should be noted that other studies may have used variants of the ECD that were truncated after the heptad repeat sequence HR1 and thus no longer contained subsequent sequences (HR2, TMD, CT).

However, there is a discrepancy in the literature regarding the effect of proline residues on spike stability. This is also evident in the data sets shown in Fig. 3, which are represented by corresponding symbols. The native variant of the S protein is represented by filled symbols, while the S proteins with 2 and 6 proline residues respectively are depicted by half-filled and empty symbols. As can be seen from the figure, it is not possible to draw reliable conclusions regarding the stabilization of the ECD by proline residues. Some studies have found stabilization by six proline residues^36,37,47^ while others have not detected stabilization by six proline residues when compared to S proteins with two or no proline residues^40,45,46^.

However, even with the 6 P variants, a reduction in Tm_1_ is evident for the Omicron variants BA.1 and or BA.2 compared to wt Tm_1_^38,40,45^. This indicates that despite the artificially introduced protein mutations, the influence of natural mutations on the thermal behavior of the spike proteins of the VoCs remains and is detectable. This is further confirmed by an analysis of the thermal behavior of the RBD.

## Thermal behavior of the RBD

**Fig. 5:**
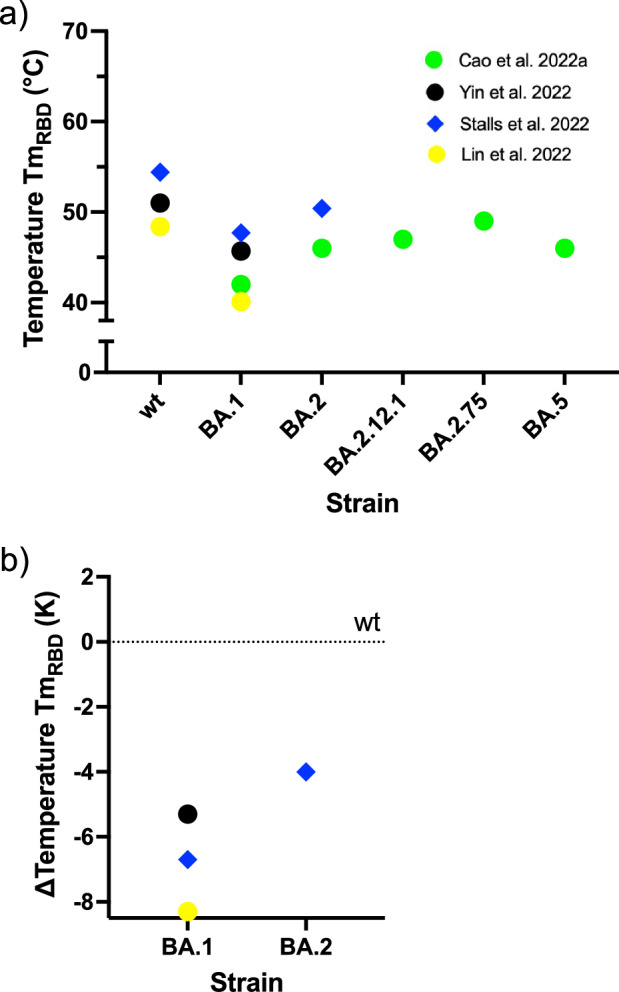
The thermostability of the RBD of spike protein variants measured by DSF. **a** The melting temperature Tm_RBD_ is shown. **b** Differences ΔTm_RBD_ between Tm_RBD_ of the wt and the Tm_RBD_ values of the measured VoCs of the individual selected study.

Few studies have measured the thermal stability of the RBD of the wt of Omicron variants using DSF. In contrast to the ECD trimer, only one temperature inflection point, Tm_RBD_, was found (Fig. 5a). The range of Tm_RBD_ is similar to that of Tm_1_ of the ECD trimer. Fig. 5b shows the difference in Tm_RBD_ of the Omicron variants compared to the Tm_RBD_ of the wt for each study. As before, there is a marked decrease in thermal stability for BA.1 and BA.2. For BA.1, the decrease in ΔTm_RBD_ is between 5 and 8 K. Cao et al. ^41,42^ has measured the Tm_RBD_ of the RBD for different Omicron variants (Fig. 5a), but not for the wt. Therefore, the study could not be included in Fig. 4b. However, the data from Cao et al. ^41,42^ (see Fig. 5a) indicate that the decrease in the thermal stability of the RBD of BA.1 was no longer observed with subsequent Omicron variants. Again, the survey indicates that there is also a thermal transition in the RBD, which can be identified with Tm_1_ of the whole ECD.

## Surface charge evolution and structural dynamics in Omicron spike variants

Despite the advantageous impact of an increased positive surface potential on the initial binding of SARS-CoV-2 to the host cell^48,49^ (see also “Introduction”), the present study shows that the elevated positive surface potential of the S spike protein of the original Omicron variant BA.1 compared to the wt was not further increased in the subsequent Omicron variants, including the more recent ones, for example JN1, KP.2, KP.3. We even observed a slight reduction in the number of charged amino acids in the S1 domain. This reduction did not significantly change the pattern of the positive surface potential in the distal part of the ECD known from BA.1 and BA.2. In the stem region (S2 subunit), the neutral surface potential of the wt strain remained almost unchanged during the genesis of the various Omicron variants. It is known that the highly conserved sequence of the stem region of the S protein reflects its functional relevant stability. This is also known for the stem region of spike proteins of other viruses, e.g., of hemagglutinin of influenza A viruses^50^.

One potential explanation for the absence of a further increase of the surface potential of the ECD of Omicron variants is that, as has been observed in other proteins^26,27,30^, a further increase may have a destabilizing effect on the ECD. This could impact the metastability of the prefusion conformation of the ECD and could impair its function of mediating the fusion of the viral envelope with the plasma or the endosomal membrane. To address this hypothesis, we reviewed studies on the thermal stability of the ECD and RBD across various viral variants, including the wt strain and other VoCs.

Despite the variability of the reported data, the collective evidence points to the presence of two distinct thermal transitions of the ECD: Tm_1_ between 45 °C and 55 °C, associated with local conformational changes, and Tm_2_ between 64 °C and 70 °C reflecting a global change of the ECD structure. Indeed, DSC measurements^35–37^ and their enthalpy values provide evidence that the intense peak characterized by Tm_2_ reflects a structural change of the entire ECD, most likely its denaturation, i.e., unfolding. The Tm_2_ values for the S spike protein of BA.1 and BA.2 were not reduced compared to the wt strain suggesting that the stability of the ectodomain is preserved or even enhanced, at least for these two VoCs (data for other variants are not available). This is supported by the DSC data of Zhou et al., which showed not only an increase in Tm_2_ but also a moderate increase in enthalpy for BA.1 compared to wt^37^. It can therefore be concluded that the increased positive surface potential and the associated mutations do not lead to a reduction in the thermal stability of the S protein of BA.1. This finding is consistent with the results of independent studies showing a more compact S protein structure in Omicron, suggesting tighter monomer packing^40,45,51,52^.

In contrast to the pronounced peak characterized by Tm_2_, the peak measured at the lower temperature Tm_1_ is much less pronounced. A number of studies have indicated that the Tm_1_ transition does not reflect a change in the overall ECD conformation, but rather a local structural change within the ECD. The melting temperature of the RBD Tm_RBD_ is within the same range as that of Tm_1_ of the ECD^53^ (compare Figs. 3a and 5a). This suggests that Tm_1_ is related to changes in the structure of the RBD which could affect the up-down transition of the RBD within the trimer. Using cryo-EM Edwards et al.^53^ demonstrated that Tm_1_ of the ECD reflects a conformational change of the RBD, most likely favoring its up conformation. Juraszek et al.^35^ reported that the transition at Tm_1_ measured by DSC almost disappeared when the down conformation of the RBD was stabilized by mutations. Indeed, very recently, DSC revealed that the Tm_1_ peak, but not the Tm_2_ transition, was absent when the RBD of the wt ECD was locked in the down conformation by disulfide bonds^36^. The association of Tm_1_ with the up-down transition of the RBD is supported by the findings of Javanmardi et al.^38^, who generated a series of chimeric proteins comprising the S ectodomain of the wt Wuhan strain. These proteins were constructed by replacing the NTD, RBD, and S2 domains with those of BA.1 and BA.2, respectively. The authors have presented evidence that the lowered Tm_1_ of BA.1 and BA.2 compared to the wt is due to the RBD of BA.1 and BA.2^38^. The aforementioned stabilization of the down conformation does not necessarily contradict the reduced Tm_1_ of the up-down of the RBD. Structural studies have indicated that the BA.1 ECD is primed for a transition into the 1-RBD-up state^45,51^ but not into the 2- or 3-up state of the trimer. One potential explanation for this phenomenon is an increased electrostatic repulsion between the distal regions of the ECD monomers of the Omicron variants. A reduction in repulsion could be achieved by an upstate, which may be a preferred configuration. Notably, binding of HS to the RBD has also been shown to support and stabilize the transition to an RBD-up state facilitating interaction with the cellular receptor^14,54^. Although we can see that the change in Tm_1_ observed for BA.1 and BA.2 correlates with the increased surface potential, the results do not allow us to conclude that this is causally related, other mutations not considered here may also be responsible. Furthermore, it is not possible to draw any conclusions about recent Omicron variants due to the lack of investigations in this regard.

## Conclusion

The present analysis shows that the stability of the ECD of the Omicron VoCs under investigation is only slightly affected, if at all, by the increased positive surface potential in the ECD. It also shows that the numerous mutations not considered in this study do not exert a destabilizing influence on the ECD structure. Although it was found that individual mutations in themselves have a destabilizing effect, this is obviously compensated for by epistasis, i.e., by other mutations^55,56^. For this reason, destabilizing mutations have the potential to manifest themselves in subsequent variants, as they can have a positive influence, e.g., on the binding of ACE2, and thus on the fitness of the virus^57^. In this sense, an increased surface potential and its advantage for binding to the host cell can be maintained despite a potentially negative influence on the stability of the ECD.

Nevertheless, the question remains why, in view of the aforementioned advantages of a positive surface potential of the ECD for interaction with the host cell, no further increase in this potential was seen with Omicron VoCs following BA.1. Two potential explanations for this phenomenon would be a destabilization of the ECD, which could no longer be compensated for, and/or an intense local attachment to the negatively charged surface of the host cell, which could impair lateral ‘surfing’ and thus the interaction with ACE2. Evolution could therefore have achieved a balance between optimizing binding to the host cell and at the same time preserving the structural stability of the ECD.

An impressive example of epistasis is the preservation of the stability of the nucleoprotein of influenza viruses during the course of their evolution^58^. Mutations that promote escape from the host immune system had in themselves a destabilizing effect on the protein structure. However, previous mutations in the nucleoprotein compensated for this effect and preserved the stability of the protein structure. The stability was characterized in this study via the melting temperature measured by CD^58^. However, the example also showed that epistasis sets limits for the evolution of proteins: Destabilizing mutations can only manifest themselves if stabilizing mutations have occurred previously (or simultaneously).

The importance of maintaining of structural stability is further emphasized when considering that the pH of the nasal cavity’s airway-facing surface between 5.5 and 6.5^59–61^. In such an environment, the positive surface potential of the S protein is further accentuated^62^. This highlights the delicate balance between functional adaptation and structural integrity in the ongoing evolution of SARS-CoV-2.

Notably, in this study on the stability of the S ECD of SARS-CoV-2, we focused exclusively on the role of charged amino acids. While our findings suggest that mutations affecting the number of charged amino acids, as well as other mutations, do not significantly affect the overall stability of the envelope glycoprotein, this approach does not provide a complete picture of the factors influencing ECD stability. We did not consider the relevance of disulfide bonds and glycosylation of the ECD. Several studies have demonstrated their importance for both the proper folding and stability of the ECD structure. Additionally, glycosylation functions as a protective shield around the ECD, impairing degradation by proteases and recognition by the immune system (see, for example, Dadonaite et al.^63^). However, the disulfide bonds and N-glycosylation sites in the wt ECD^64,65^ are highly conserved among the VoCs although shorter glycans, a reduced sialic acid content at several N-sites and site-specific suppression of glycosylation have been observed for Omicron variants^66–68^.

## Supplementary information

Supplementary Information

## Data Availability

This review summarizes data, which are references within the manuscript. Results from our meta-analysis are provided with the manuscript or supplementary information.
