# Supplementary material for: Balancing stability and function: impact of the surface charge of SARS-CoV-2 Omicron spike protein
Source: Npj Viruses. 2025 Apr 1;3:23. doi: 10.1038/s44298-025-00104-1 (PMC11962157; doi:10.1038/s44298-025-00104-1)
Supplement: Supplementary file 1 — Supplementary Information [file 44298_2025_104_MOESM1_ESM.pdf]

Balancing Stability and Function: Impact of the Surface Charge of SARS-CoV-2 Omicron Spike Protein

Daniel Lauster[1+], Rainer Haag[2], Matthias Ballauff[2], Andreas Herrmann[2+]

[1]Institute of Pharmacy, Biopharmaceuticals, Freie Universität Berlin, 14195 Berlin, Germany
[2]Institute of Chemistry and Biochemistry, Freie Universität Berlin, 12169 Berlin, Germany

Supplementary Information

Supplementary Table 1

Charged amino acids of the S protein ECD

Charged amino acids (D,E and R,K) of the S protein ECD of wt SARS-CoV-2 and changes in subsequent (recombinant) VoCs (colored) (Amino acid numbering in bold – RBD)

No. AA	142	145	147	152	158	180	183	213	**339**	**346**	**356**	**405**	**408**	**417**	**440**	**450**
wt	G	Y	K	W	R	E	Q	V	G	R	K	D	R	K	N	N
BA.1	G	D	K	W	R	E	Q	V	D	R	K	D	R	N	K	N
BA.2	D	Y	K	W	R	E	Q	G	D	R	K	N	S	N	K	N
BA.2.75	D	Y	E	R	R	E	Q	G	H	T	K	N	S	N	K	N
XBB.1.5	D	Y	K	W	R	E	E	E	H	T	K	N	S	N	K	N
XBB.1.16	D	Y	K	W	R	V	E	E	H	T	K	N	S	N	K	N
EG.5	D	Y	K	W	R	E	E	E	H	T	K	N	S	N	K	N
HK.3	D	Y	K	W	R	E	E	E	H	T	K	N	S	N	K	N
JN.1	D	Y	K	W	G	E	Q	G	H	R	T	N	S	N	K	D
KP.2	D	Y	K	W	G	E	Q	G	H	T	T	N	S	N	K	D
KP.3	D	Y	K	W	G	E	Q	G	H	R	T	N	S	N	K	D
BA.3	D	del	K	W		E	Q	V	D	R	K	N	R	N	K	N
BA.4	V		K	W		E	Q	G	D	R	K	N	S	N	K	N

No. AA	**452**	**460**	**478**	**481**	**484**	**493**	**498**	547	554	614	679	681	764	796	856	969
wt	L	N	T	E	E	Q	Q	T	E	D	N	P	N	D	N	N
BA.1	L	N	K	E	A	R	R	K	E	G	K	H	K	Y	K	K
BA.2	L	N	K	E	A	R	R	T	E	G	K	H	K	Y	N	K
BA.2.75	L	K	K	E	A	Q	R	T	E	G	K	H	K	Y	N	K
XBB.1.5	L	K	K	E	A	Q	R	T	E	G	K	H	K	Y	N	K
XBB.1.16	L	K	R	E	A	Q	R	T	E	G	K	H	K	Y	N	K
EG.5	L	K	K	E	A	Q	R	T	E	G	K	H	K	Y	N	K
HK.3	L	K	K	E	A	Q	R	T	E	G	K	H	K	Y	N	K
JN.1	L	K	K	K	E-K	Q	R	T	K	G	K	R	K	Y	N	K
KP.2	L	K	K	K	E-K	Q	R	T	K	G	K	R	K	Y	N	K
KP.3	L	K	K	K	E-K	E	R	T	K	G	K	R	K	Y	N	K
BA.3	L	N	K	E	A	R	R	T	E	G	K	H	K	Y	N	K
BA.4	R	N	K	E	A	Q	R	T	E	G	K	H	K	Y	N	K

Color	Meaning
(red)	Negatively charged aa added (D or E
(orange)	Negatively charged aa removed (D or E)
(blue)	Positively charged aa added (R or K)
(green)	Positively charged aa removed (R or K)

Sequences are from

wt - https://www.ncbi.nlm.nih.gov/protein/YP_009724390.1
BA.1* - Slovenia/178530/2022
BA.2* - Norway/1831/2022
BA.2.75* - Croatia/HZJZ_1619/2022
XBB.1.5* - Denmark/DCGC-662916/2023
XBB.1.16* - Denmark/DCGC-662929/2023
EG.5* - Austria/AGES-AZ-1662675/2023
HK.3* - France/OCC-CHU-TLS-6004314441/2023
JN.1 - https://outbreak.info/situation-reports?xmin=2023-12-22&xmax=2024-06-22&pango=JN.1;
KP.2 - https://outbreak.info/situation-reports?xmin=2023-12-22&xmax=2024-06-22&pango=KP.2
KP.3 - https://outbreak.info/situation-reports?xmin=2023-12-22&xmax=2024-06-22&pango=KP.3;
BA.3 - https://outbreak.info/situation-reports?xmin=2023-12-11&xmax=2024-06-11&pango=BA.3
BA.4* - Pakistan/NIH-B69-S5/2022

*BA.1, BA.2, BA.2.75, XBB.1.5., XBB.1.16, EG.5, HK.3 BA.4 are taken from https://nextstrain.org/ncov/gisaid/global/6m?s=hCoV-19/

Supplementary Table 2

The thermal stability of the ECD and of the RBD of the S-protein

Overview on studies of the thermal stability of the trimeric ECD and of the RBD of S protein of SARS-CoV-2 strains. Only one study has used Differential Scanning Calorimetry (DSC) for the thermal stability of ECD (Zhou et al. 2022). All other data shown were obtained by Differential Scanning Fluorimetry (DSF). Fluorescence was measured either intrinsically (i) or by using the dye SYPRO Orange (S). Depending on the study using DSF temperatures of up [1]to three inflection points have been measured ($Tm_1 < Tm_2 < Tm_3$).

Ref	Object		wo/w 2P, 6PP			pH	Method		Scan rate K/min	Temperature (°C) (for DSF $Tm_1 < Tm_2 < Tm_3$)												
							DSC	DSF S,i		wt	α	β	γ	ε	δ	Omicron BA.						
	ECO	RBD	woP	2P	6P											1	2	2.12.1	2.13	2.75	3	4/5
Zhou 2022	+				+		x		1	63.8						64.7						
Juraszek 2021	+			+			x		0.9	49<br>64												
Cao 2022	+				+	7.4		S	1							63	60	61		66		61
	+				+	5.5										63	60	60		62		60
		+		n.a.		7.4										42	46	47		49		46
Cao 2022	x					7.4		S	1							64	59	62	61		62	65
Yin 2022	+			x				S	3.6	52.9<br>71.5						46.6<br>71.5						
		+		n.a.						51.0						45.7						
Xu 2022	+			x		7.4		S	3.6							44.5<br>70.3						
	+				x					52.5<br>70.9							47.4<br>66.8					
Hsieh 2020	+			+		8.0		S	4.4	46												
	+				+					51												
Wang 2022	+				+	7.2		S	3.6	61.2						67.2						
Javanmardi 2022	+				+	7.5		S	4.5	52<br>69.1	49.8<br>69.1	47.5<br>74.0	51.5<br>70.7	52<br>64.3	51.5<br>66.0	44.8<br>71.1	45.9<br>68.7					
Lin 2022		+				7.5		S	0.93	48.4						40.1						
Guo 2022	+				+	7.0		i	30	52,6<br>68.0<br>76,4						53.8<br>64.6<br>71.6	49.6<br>64.6<br>72.4					
Gobeil 2021	++		+			7.4		i	30	51.2<br>64.0<br>74.8												
	++			+						53.2<br>65.4<br>74.5												
	+		+							55.3<br>65.8<br>75.3												
Gobeil 2022	+		+			7.4		i	30	55.4<br>66.8<br>75.3					55.4<br>65.5<br>75.3	51.3<br>65.3<br>74.7						
	+			+												49.0<br>66.8<br>75.0						
Stalls 2022	+		+			8.0		i	30	55.5<br>65.9<br>75.5						50.8<br>67.4<br>77.0	54.4<br>67.5<br>76.0					
		+		n.a.						34.4						47.7	50.4					
Rutten 2024	+				+	7.4		S	0.9	48.4<br>61.8												

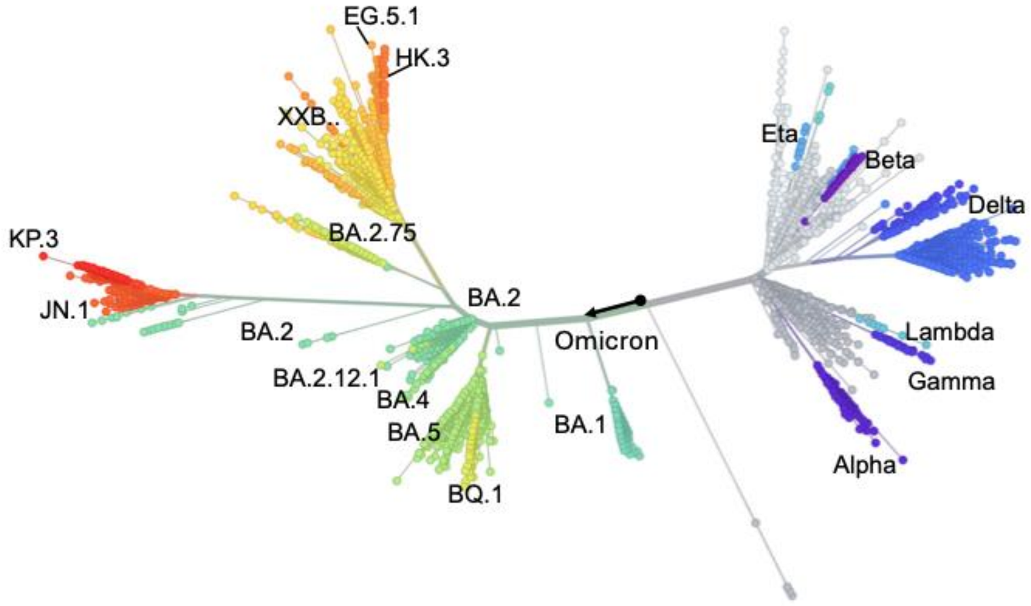

Supplementary Figure 1
Phylogeny of SARS-CoV-2 variants used for analysis
The lineage tree was generated via nextstrain.org/sars-cov-2[2].

(1) Dadonaite, B.; Ahn, J. J.; Ort, J. T.; Yu, J.; Furey, C.; Dosey, A.; Hannon, W. W.; Vincent Baker, A. L.; Webby, R.; King, N. P.; et al. Deep mutational scanning of H5 hemagglutinin to inform influenza virus surveillance. *bioRxiv* **2024**, 2024.2005.2023.595634. DOI: 10.1101/2024.05.23.595634.
(2) Hadfield, J.; Megill, C.; Bell, S. M.; Huddleston, J.; Potter, B.; Callender, C.; Sagulenko, P.; Bedford, T.; Neher, R. A. Nextstrain: real-time tracking of pathogen evolution. *Bioinformatics* **2018**, *34* (23), 4121-4123. DOI: 10.1093/bioinformatics/bty407 (acccessed 9/19/2024).
